# Case of a Painful Affection of the Face Cured by Electricity

**Published:** 1786

**Authors:** Robert Blunt

**Affiliations:** Surgeon at Odiham, in Hampshire


II. *Cafe of a painful Affection of the Face cured by Electricity. By Mr.* Robert Blunt, *Surgeon at*

*at Odiham, in Hampſhire. Communicated in a Letter to* William Wright, *M. D. F. R. S. and by him to Dr.* Simmons.

MRS. Webb, aged ſixty-three years, was, on the 3d of January, 1783, ſuddenly ſeized with a violent pain in her right temple, which continued during the ſpace of about a minute. This pain came on, without any apparent cauſe, while ſhe was dreſſing herſelf; but ſhe felt no more of it till the next morning, when it returned with as great acuteneſs as before, and in the afternoon (Jan. 4) ſhe thought proper to apply to me.

Her pulſe at this time was ſoft and regular, and ſhe had no ſymptom that could tend to throw any light on the cauſe of her complaint; being apparently in a ſtate of perfect health, ſuch as ſhe had enjoyed for a conſiderable time before. But as ſhe was rather of a full habit, I was of opinion that moderate evacuations would be proper for her; and accordingly I adviſed her to loſe eight ounces of blood, and the next day to take a gentle purgative. In the courſe of that day (Jan. 5th) the pain returned ſeveral times, and I was preſent during one of the paroxyſms, which laſted nearly five minutes. As ſoon as ſhe was able to ſpeak, ſhe deſired desired her servant to hold her head as hard as she could, appearing at the same time, from the contortion of her features, to be in great agony. As soon as the paroxysm was over, she got up from her chair, and said she was perfectly free from pain.

From this period, till the 12th of February, she every day felt more or less of the complaint, and in general experienced several returns of it each day. If she wiped her mouth with an handkerchief, or opened her mouth to speak or eat, the pain would oftentimes come on instantaneously; so that, although she had a desire for food, she was deterred from taking a proper supply of nourishment, and soon became much reduced.

In this case different antispasmodics were recommended and tried, without the least good effect. Blisters behind the ears, and on the back, afforded her no relief. Leeches applied to the temples were equally inefficacious; and æther, though rubbed frequently on the part affected, did not seem in the least degree to mitigate the pain. As the complaint seemed to be perfectly analogous to that described by the late Dr. Fothergill *, the extract of hem-

* Medical Observations and Inquiries, Vol. V. page 129.

lock, which he had mentioned as an efficacious remedy in fimilar cafes, was prefcribed on his authority, and had a fair trial, but with no better fuccefs than the other medicines fhe had taken.

In this diftrefsful fituation I recommended to her electricity, and fhe readily confented to give it a trial. For this purpofe fhe came to my houfe on the 12th of February in a poft-chaife, as the weather was then very cold, and fhe lived at the diftance of two miles from Odiham. I electrified her twice that day, during about twenty minutes each time; the firft time drawing fparks fimply, and the fecond time drawing fparks, and paffing a few flight fhocks through the part which had been uniformly the feat of the pain. As foon as the fecond trial was over, having a great defire for food, and thinking herfelf better, fhe ventured to eat fome roafted veal, and, to the aftonifhment of herfelf and fome of her friends who were with her, felt not the leaft inconvenience from the complaint. She had a flight return of the pain, however, that evening on her way home; but paffed a good night, and found herfelf much better the next morning, (February 13) when fhe took fufficient nourifhment, ment, but in the courſe of the day had a few very ſlight returns of the pain.

On the 14th electricity was repeated, and ſhe found herſelf ſtill better. On the 16th it was tried again, and for the laſt time, as a total ceſſation of the pain did not ſeem to render any farther repetition of it neceſſary; and ſince that period ſhe has remained free from the complaint, and is now in perfect health.

This is the only caſe of the kind that has fallen under my obſervation; but as this painful affection ſeems fortunately to be one of thoſe complaints which occur but ſeldom, (the late Dr. Fothergill having been able to recollect only about ſixteen * caſes of it in the courſe of his long and extenſive practice) I flatter myſelf you will think the preſent inſtance, though a ſolitary one, of its having been ſo ſpeedily removed by electricity, worthy of being recorded.

*Odiham, Hants,*
January 4, 1786.

* Med. Obſ. and Inq. Vol. V. p. 130 and 142.

